# Fabrication of an antibody-aptamer sandwich assay for electrochemical evaluation of levels of *β*-amyloid oligomers

**DOI:** 10.1038/srep35186

**Published:** 2016-10-11

**Authors:** Yanli Zhou, Huanqing Zhang, Lantao Liu, Congming Li, Zhu Chang, Xu Zhu, Baoxian Ye, Maotian Xu

**Affiliations:** 1 Henan Key Laboratory of Biomolecular Recognition and Sensing, College of Chemistry and Chemical Engineering, Shangqiu Normal University, Shangqiu 476000, P. R. China; 2 College of Chemistry and Molecular Engineering, Zhengzhou University, Zhengzhou 450001, P. R. China

## Abstract

Amyloid *β*-peptide (A*β*) in its oligomeric form is often considered as the most toxic species in Alzheimer’s disease (AD), and thus A*β* oligomer is a potentially promising candidate biomarker for AD diagnosis. The development of a sensitive and reliable method for monitoring the A*β* oligomer levels in body fluids is an urgent requirement in order to predict the severity and progression at early or preclinical stages of AD. Here, we show a proof of concept for a sensitive and specific detection of A*β* oligomers by an antibody-aptamer sandwich assay. The antibodies of A*β* oligomers and a nanocomposite of gold nanoparticles with aptamer and thionine (aptamer-Au-Th) were used as the recognition element and the detection probe for specifically binding to A*β* oligomers, respectively. The electrochemical signal of Th reduction could provide measurable electrochemical signals, and a low limit of detection (100 pM) was achieved due to the signal amplification by high loading of Th on the gold nanoparticles. The feasibility of the assay was verified by test of A*β* oligomers in artificial cerebrospinal fluid. The proposed strategy presents valuable information related to early diagnosis of AD process.

Alzheimer’s disease (AD) is the most prevalent progressive dementia marked by memory loss, cognitive decline, behavioral and physical disability, and significant and irreversible brain damage^1^,^2^. With a steady increase in the aging population, AD has become a serious social problem. Although some agents have been utilized to alleviate the symptoms of AD patients, there are no powerful therapeutic drugs for radical cure of AD. Thus, early diagnosis of AD is an urgent case for preventative and therapeutic treatment.

Amyloid *β*-peptide (A*β*) is the major component of the senile plaques that are one of two classical pathological hallmarks of AD^3^. The amyloid cascade hypothesis enunciates that an increased A*β* aggregation produces firstly to the formation of A*β* oligomers, then fibrils, and ultimately to plaques. It is now widely accepted that the diffusible A*β* oligomers, rather than mature A*β* fibrils and small A*β* monomers, have also been highlighted as the most neurotoxic form^4^. The neurotoxic increase of the A*β* oligomers could be explained by an increase in the number of toxic *β*-sheets per total mass of A*β*. The levels of A*β* oligomers in cerebrospinal fluid (CSF) of AD patients are higher than that of normals^5^. The levels of A*β* oligomers in CSF are efficacious for predicting the severity and progression at early or preclinical stages of AD. Therefore, A*β* oligomers are now considered not only as diagnostic markers but also as therapeutic targets of AD^6^. However, the detection of A*β* oligomers is a great challenge due to their characteristics of instability and transience to produce heterogeneous mixtures during the analysis process.

Electrochemical sensors^7^,^8^,^9^, surface plasma resonance sensors^10^,^11^, and fluorescent sensors^12^,^13^,^14^,^15^,^16^,^17^,^18^,^19^,^20^, and surface-enhanced Raman spectroscopy^21^ to measure A*β* oligomers have been designed based on the high binding affinity of peptides, proteins, antibodies and small molecules toward A*β* oligomers^22^. Although a low detection of these assays was obtained, a drawback of above fabricated assays was their low specificity owing to the interference and nonspecific adsorption from body fluids. Detection of A*β* oligomers by large-scale instruments, such as mass spectrometry could achieve the high reproducibility and sensitivity^23^,^24^. The inherent shortcomings of expensive instruments and complex operation still existed. Enzyme-linked immunosorbent assay (ELISA) technique is a reliable method for analyzing A*β* oligomers and is easy to realize miniaturization. ELISA, which utilized two approaches based on conformation-specific and sequence-specific antibodies, have been widely reported for detection of A*β* oligomers. The first strategy suffered from the interferences of other oligomeric proteins, such as prion, *α*-synuclein, polyglutamate, and several heat shock proteins^25^. The second strategy to measure A*β* oligomers in CSF or human brain ensured the specific and sensitive detection based on using capture and (labeled) detection antibodies to recognize the A*β* oligomers^26^,^27^,^28^,^29^. However, the problem of time-consuming and requirement of expensive enzyme-lined antibodies should not be ignored. The above problems limit their applications for the routine test of the A*β* oligomers for early diagnosis of AD.

Alternatively, aptamers, which are selected through an *in vitro* selection process called selective evolution of ligands by exponential enrichment (SELEX), have the comparable binding and specificity with antibodies^30^,^31^. Importantly, aptamers are more efficient than antibodies because of the ease in conjugation to various molecules, animal-free synthesis, and improved stability^32^,^33^,^34^. Tsukakoshi *et al*. isolated A*β* oligomer-specific DNA aptamers by the combination of a gel-shift assay and a competitive screening method^35^. The selected aptamers could be potentially applied in the biological assay for AD-related research.

In consideration of the urgency of A*β* oligomer detection and the advantages of aptamers in clinical diagnosis, we introduce an antibody-aptamer sandwich assay as a sensitive, specific, and versatile electrochemical platform for protein detection. The antibodies of A*β* oligomers were used as the recognition element for specifically binding to A*β* oligomers. A nanocomposite of aptamer-Au-Th prepared by *in situ* modification of gold nanoparticles (AuNPs) with DNA aptamer and thionine (Th) was utilized as the detection probe. The electrochemical signal of Th reduction could provide measurable electrochemical signals and the signal amplification was achieved by high loading of Th on the AuNPs. Finally, the established aptamer-based electrochemical assay was successfully applied for evaluation of A*β* oligomers in artificial CSF samples.

## Results and Discussion

### Design strategy and characterization of the antibody-aptamer sandwich electrode

**Figure 1:**
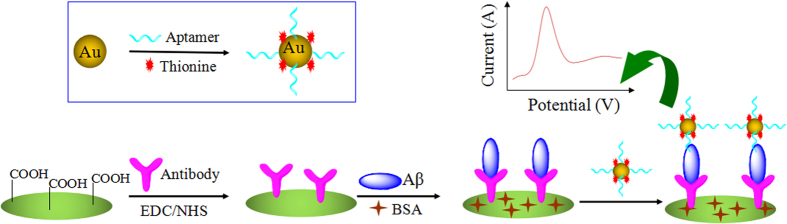
A schematic illustration of the electrochemical detection of A*β* oligomers using an antibody-aptamer sandwich assay.

**Figure 2:**
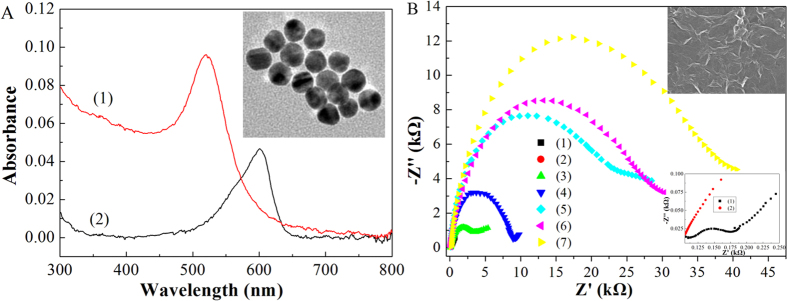
(**A**) UV-visible spectra of the aqueous solution of AuNPs (1) and aptamer-Au-Th bioconjugate (2). Inset (a) is a TEM image of AuNPs. (**B**) Nyquist plots of 10 mM [Fe(CN)_6_]^3−/4−^ in 0.1 M KCl from 0.1 MHz to 0.1 Hz at ac amplitude of 5 mV under open-circuit potential conditions, obtained at the naked GC electrode (1), and the GC electrodes after the modification of carboxyl graphene (2), the activation of NHS/EDC (3), the immobilization of antibody (4), the recognition of A*β* oligomers (5), the blocking by BSA (6), and the binding of aptamer-Au-Th bioconjugate (7). Inset (b) is a SEM image of (2). Inset (c) is the magnified Nyquist plots for (1) and (2).

Figure 1 shows the construction procedure of the antibody-aptamer sandwich electrode and its sensing mechanism for A*β* oligomers. AuNPs synthesized as a stabilizer from citrate displayed a maximal absorbance at 520 nm, which was attributed to the surface plasmon resonance of the 20 nm AuNPs verified by characterization of transmission electron microscopy (TEM) (Fig. 2A). The incubation of AuNPs with DNA aptamer for recognition of A*β* oligomers and with Th for electrochemical signal amplification produced the aptamer-Au-Th probe at an appropriate molar ratio. The prepared probe showed an absorbance peak at 600 nm, which was consistent with the reported results^36^. To further verify the modification of AuNPs, X-ray photoelectron spectroscopy (XPS) was performed to analyze the chemical composition on the surface of AuNPs (Fig. S1). The S 2p and N 1s spectrum peaks of AuNPs after modification of Th or aptamer were observed distinctly at the binding energy of 167 and 399 eV, respectively. After the modification of Th and aptamer together, both of the spectrum peaks increased to some extent. The above results indicated that the aptamer-Au-Th probe was successfully prepared.

The glassy carbon (GC) electrode was fabricated by use of carboxyl graphene as the substrate because of not only its rapid electron transfer process but also its ability to immobilize the antibody onto the electrode surface. The image of scanning electron microscopy (SEM) for the carboxyl graphene-modified GC electrode demonstrated that a wrinkled texture of graphene sheets was formed on the electrode surface (inset b of Fig. 2B). The A*β* oligomers could be specifically recognized by the antibody tethered to the assay, which was followed by the binding of aptamer-Au-Th bioconjugates. The selected aptamers could bind A*β* oligomers specifically because of their high affinity (dissociation constant was estimated as 25 nM)^35^. The sandwich assay was established and the electrochemical reduction of Th was utilized for the quantitative detection of A*β* oligomers in 0.1 M phosphate buffer solution (PBS, pH 7.4). As we know, this is the first electrochemical assay about determination of the level of AD biomarker based on the binding with DNA aptamer. The formed assay was expected to have high sensitivity and selectivity because of the signal amplification by aptamer-Au-Th probe and the high specificity of antibody and aptamer, respectively.

Electrochemical impedance spectroscopy was employed to study the interface properties of the electrode surface during the fabrication procedure. The semicircle diameter at higher frequencies of the Nyquist plot is equal to the electron transfer resistance (*R*_et_), which corresponds to the electron-transfer-limited process. Figure 2B illustrated the typical Nyquist plots for the modified electrodes in 0.1 M KCl containing 10 mM [Fe(CN)_6_]^3−/4−^. The *R*_et_ at the GC electrode could be estimated to be 76 ± 2 Ω. After casting the carboxyl graphene on the GC electrode, the *R*_et_ decreased nearly to zero, demonstrating that the graphene layer promoted the electron transfer process between the electrode surface and electrolyte. The *R*_et_ increased dramatically to 2.38 ± 0.05 kΩ, 6.24 ± 0.18 kΩ, 15.37 ± 0.36 kΩ, 18.46 ± 0.54 kΩ, and 23.15  ± 0.79 kΩ after the activation of NHS/EDC, the immobilization of antibody, the recognition of A*β* oligomers, blocking by bovine serum albumin (BSA), and binding of aptamer-Au-Th bioconjugate, respectively. The increase in *R*_et_ results from the hindered pathway of electron transfer because the most organic and biological molecules are poor electrical conductors and produce hindrance to electron transfer. These results verified the fabrication process of the antibody-aptamer sandwich assay.

### Voltammetric determination of A*β* oligomers

**Figure 3:**
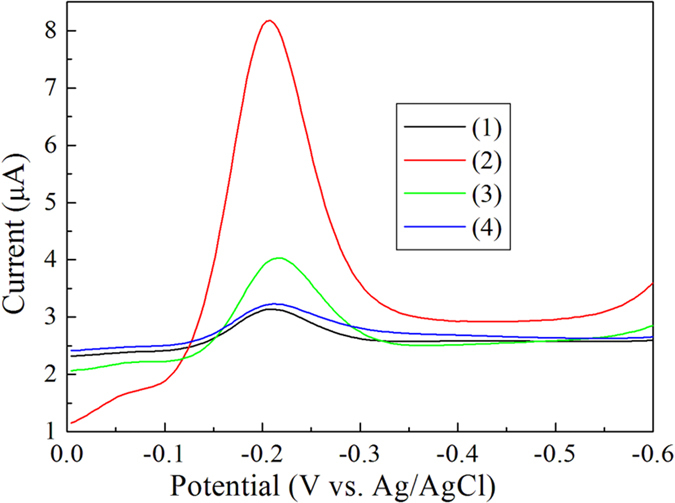
DPV of the antibody-aptamer sandwich assay in 0.1 M PBS (pH 7.4). In the absence (1) and presence (2) of 20 nM A*β* oligomers after blocking of BSA. Curve 3 is the absence of 20 nM A*β* oligomers before blocking of BSA. Curve 4 is the presence of 20 nM A*β* oligomers without the immobilization of antibody.

Figure 3 shows a comparison of differential pulse voltammetry (DPV) using the antibody-aptamer sandwich assay in the presence and absence of the A*β* oligomers in 0.1 M PBS (pH 7.4). The reduction peak current of Th at −0.20 V for the control experiment showed the non-specific binding of the aptamer-Au-Th bioconjugates over the electrode surface before blocking of BSA. The signal decreased after blocking of BSA due to the less non-specific adsorptions. Obvious increase of reduction peak current of Th in the presence of A*β* oligomers was observed, which resulted from the recognition of A*β* oligomers by both antibody and aptamer. To further verify the antibody recognition, the control experiment without the immobilization of antibody was also carried out and just a very small peak current was obtained because of the bioconjugate adsorption on the electrode surface. The results indicated that the electrochemical assay could be used for the detection of A*β* oligomers.

**Figure 4:**
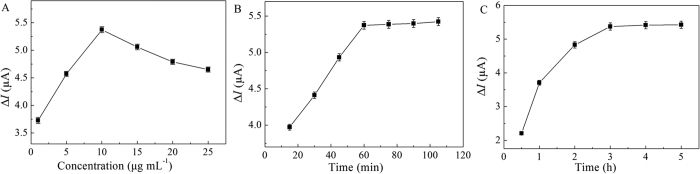
Effect of antibody concentration (**A**), incubation time of A*β* oligomers (**B**), and incubation time of aptamer-Au-Th bioconjugate (**C**) for detection of 20 nM A*β* oligomers in 0.1 M PBS (pH 7.4) using the antibody-aptamer sandwich assay.

To optimize analytical conditions for voltammetric assay, experimental parameters including antibody concentration, incubation time of A*β* oligomers, and incubation time of aptamer-Au-Th bioconjugate were studied. The increased current Δ*I* = *I*_p_ − *I*_0_ was utilized as the binding parameter of A*β* oligomers, where the *I*_p_ and *I*_0_ was the peak current in the presence and absence of A*β* oligomers, respectively. The effect of antibody concentration on the peak current of Th reduction was investigated from 1 to 25 μg mL^−1^ as shown in Fig. 4A. The Δ*I* value gradually increased with increasing concentration from 1 to 10 μg mL^−1^, and then it began to decrease as the concentration increased over 10 μg mL^−1^ due to the hindered pathway of electron transfer by the high loading of antibody molecules. Thus, the optimum antibody concentration was used for subsequent experiments. The effect of incubation time of A*β* oligomers (Fig. 4B) and aptamer-Au-Th bioconjugate (Fig. 4C) on the detection of A*β* oligomers was then performed, and the Δ*I* value reached the steady state over 60 min and 3 h incubation time, respectively. Hence, the incubation time of A*β* oligomers and aptamer-Au-Th bioconjugate was determined to be 60 min and 3 h, respectively.

**Figure 5:**
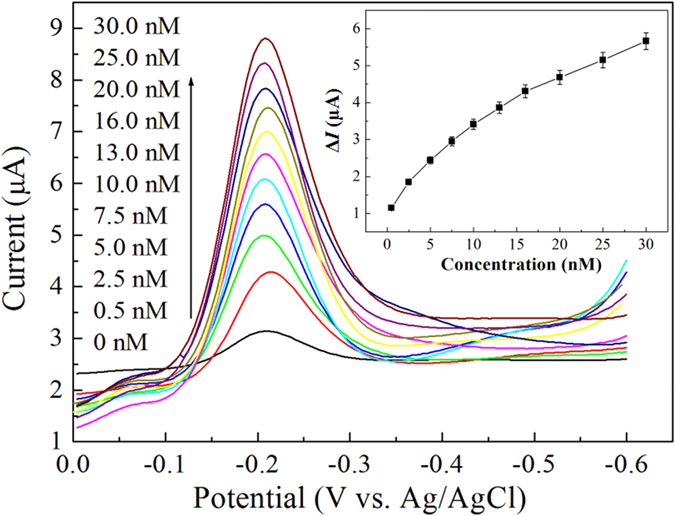
DPV curves of an increasing concentration of A*β* oligomers in 0.1 M PBS (pH 7.4) using the antibody-aptamer sandwich assay. Inset is the linear calibration plot of (*I*_p_ − *I*_0_) value *vs*. the concentration of A*β* oligomers.

The antibody-aptamer sandwich assay was examined under the optimum conditions for the determination of A*β* oligomers. In Fig. 5A, the reduction peak current increased upon the increase of the concentration of A*β* oligomers. As shown in Fig. 5B, the Δ*I* value was linearly related to the concentration of A*β* oligomers within the range of 0.5–30 nM (Δ*I* = 0.159*c* + 1.551) with a correlation coefficient of 0.993. The limit of detection (LOD), estimated from 3σ of the baseline signals, was approximately 100 pM for the A*β* oligomers. The obtained LOD of this assay could be comparable to that of other methods such as, ELISA, SERS, MS, SPR, electrochemical and fluorescent sensor^7^,^8^,^9^,^10^,^11^,^12^,^13^,^14^,^15^,^16^,^17^,^18^,^19^,^20^,^21^,^22^,^23^,^24^,^25^,^26^,^27^,^28^,^29^. The high sensitivity of the proposed assay might be explained by the highly efficient signal amplification by the aptamer-Au-Th bioconjugate. Importantly, this method obviates the utilization of enzyme-linked antibody and the operation of complex and expensive instruments. The analytical properties, advantages, and disadvantages of these techniques are summarized in Table S1. Moreover, the physiological level of A*β* in normal human CSF is about 1–2 nM, and the concentration is lower than that of AD patients^3^. Thus the proposed method is promising to detect the A*β* oligomers in body fluids.

The performances of the developed electrochemical assay for the detection of A*β* oligomers, including repeatability, reproducibility and stability, were also studied by testing the current response of DPV to 20 nM A*β* oligomers in 0.1 M PBS (pH 7.4). The relative standard deviation (RSD) was 2.9% for 9 successive assays. The fabrication reproducibility was assessed at seven different antibody-aptamer sandwich assay prepared under the same conditions, and the RSD was 6.3%. After being stored in refrigerator (4 °C) for two weeks, 91% of its initial current response was retained. The above results of the modified electrode demonstrated an acceptable stability and reliability, which was comparable with that of the reported assays.

### Selectivity of the assay

**Figure 6:**
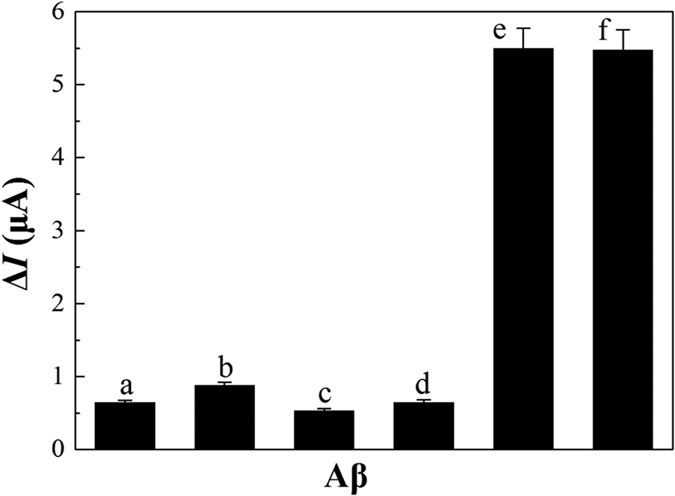
Selectivity of the antibody-aptamer sandwich assay in 0.1 M PBS (pH 7.4) containing 20 nM A*β*. (**a**) A*β*_1–42_ monomers, (**b**) A*β*_1–40_ monomers, (**c**) A*β*_1–42_ fibrils, (**d**) A*β*_1–40_ fibrils, (**e**) A*β*_1–42_ oligomers, (**f**) A*β*_1–40_ oligomers.

In order to evaluate the selectivity of the system for the detection of A*β* oligomers, the fabricated assay was used to test the A*β* molecules including A*β*_1–42_ monomers, A*β*_1–40_ monomers, A*β*_1–42_ oligomers, A*β*_1–40_ oligomers, A*β*_1–42_ fibrils, and A*β*_1–40_ fibrils, under the identical conditions. Figure 6 shows the comparison of the electrochemical response of the above A*β* molecules. It was clear that A*β* monomers and A*β* fibrils had minor influences for the detection of A*β* oligomers owing to high recognition ability of antibodies to A*β* oligomers. In addition, the similar current response for both of A*β*_1–42_ oligomers and A*β*_1–40_ oligomers indicated that the total A*β* oligomers were detected by the electrochemical assay. Therefore, the outstanding advantages of the assay with high selectivity and stability could be potentially applicable in real samples of body fluids.

### Application in CSF samples

To demonstrate the viability of this technique, the total A*β* oligomers were analyzed in real samples of artificial CSF, as illustrated in Table 1. The standard addition method analyzed by calibration curve and the recovery during the experiments for spiked samples were utilized to test the accuracy of the assay. The data in Table 1 showed the acceptable recovery, which indicated the validity of the electrochemical analysis for the A*β* oligomers in CSF samples using the fabricated assay. Thus, the simplicity, high sensitivity and selectivity of the antibody-aptamer sandwich assay make it as a new potential platform for the detection of A*β* oligomers for real samples in body fluids of AD patients.

## Conclusions

In summary, this study demonstrates the effective determination of A*β* oligomers based on the antibodies of A*β* oligomers and a nanocomposite of aptamer-Au-Th as the recognition element and the detection probe, respectively. Compared with the known method for detection of A*β* oligomers, the fabricated electrochemical assay offers some advantages: (i) high sensitivity due to the signal amplification by high loading of Th on the AuNPs, (ii) high specificity owing to the high specific recognition of antibodies and DNA aptamers with A*β* oligomers, and (iii) obviation of the utilization of enzyme-linked antibody and the operation of complex and expensive instruments. All of these factors make the antibody-aptamer sandwich assay as an ideal platform for A*β* oligomers. We believe that our work represents a significant step forward to the routine detection of A*β* oligomers and would be valuable for the early diagnosis of AD.

## Methods

### Chemicals and materials

A*β*(1–40) and A*β*(1–42) were purchased from DgPeptides Co., Ltd (Shanghai, China) with purity of >95%. The aptamer sequence of A*β* oligomers (5′-HS-GCCTGTGTTGGGGCGGGTGCG) was screened out by Tsukakoshi *et al*.^35^, and were synthesized by Sangon Biotech Co., Ltd (Shanghai, China). Anti-A*β* oligomer antibody was provided by Abcam (Cambridge, England). Chloroauric acid trihydrate (HAuCl_4_·3H_2_O), Th, NHS (N-hydroxysuccinimide), EDC (N-(3-dimethylaminopropyl)-N′-ethylcarbodiimide hydrochloride), and BSA were purchased from Sigma-Aldrich. Carboxyl graphene dispersion was provided by the XFNANO Co., Ltd (Nanjing, China). PBS were prepared by varying the volume ratios of the solution containing Na_2_HPO_4_ and NaH_2_PO_4._ All other chemicals were purchased from commercial suppliers and used as received. Deionized water (18 MΩ cm, Milli-Q gradient system, Millipore) was used throughout the experiments.

Artificial CSF used in determination of the samples was prepared by 150 mM NaCl, 3.0 mM KCl, 1.4 mM CaCl_2_·2H_2_O, 1 mM phosphate, and 0.8 mM MgCl_2_·6H_2_O 37,38.

### Instruments

All electrochemical measurements were carried out on a CHI660D electrochemical workstation (Shanghai CH Instruments, China) using a conventional three electrode system, with a modified GC disk electrode (3.0-mm diameter) as working electrode, a platinum foil as counter electrode, and a saturated Ag/AgCl as reference electrode. UV-vis absorption characterization was performed on a UV-vis 2300 spectrometer (Shimadzu, Japan). TEM samples, which were prepared by dropping 20 μL of gold colloidal solution onto a copper grid (3 mm, 300 mesh) coated with carbon film, were examined with Tecnai G2 20 S-TWIN (FEI, America) transmission electron microscope. The surface morphology of the modified electrodes was observed by SEM (Quonxe-2000, FEI). XPS samples of AuNPs were obtained by purification at high speed centrifugation (10,000 rpm) for three times. 20 μL of gold colloidal solution was dropped onto a silicon chip, and XPS spectra were carried out on an X-ray photoelectron spectrometer (PHI-5400).

### Treatment of A*β* solution

To obtain A*β* monomers, lyophilized peptides were dissolved in 1,1,1,3,3,3-hexafluoroisopropanol (HFIP) and then the above A*β* solution (2 mg mL^−1^) was incubated overnight at room temperature^39^. The solvent of HFIP was evaporated off by treatment of N_2_ gas and the A*β* was redissolved in dimethylsulfoxide. The prepared A*β* monomer solution (11.5 μM) was stored at −20 °C as stock solution. The A*β* oligomers and fibrils were obtained by incubation of the A*β* monomer solution (11.5 μM) at 37 °C at dark for 24 h and 72 h, respectively.

### Preparation of aptamer-Au-Th bioconjugate

The citrate stabilized^36^ 20 nm AuNPs were synthesized as follows: 3.75 mL trisodium citrate solution (1%) was added to a boiling HAuCl_4_ solution (0.01%, 250 mL) with rapid stirring, and then the mixture color changed from pale yellow to deep purple within 2 min. The solution was kept boiling, stirred for 15 min, after which, the AuNPs solution was cooled to room temperature. 7.0 mL of saturated Th was added into 35 mL of AuNPs solution, and then the resulting solution was mixed for 24 h. The above solution was concentrated to 5.0 mL by centrifugation (8, 000 rpm) for 15 min, followed by washing and resuspension with 0.1 M PBS (pH 7.4). 45 μL of aptamer aqueous solution (100 μM) was then added into the 5.0 mL of Au-Th solution and incubated for 4 h (140 rpm) at room temperature. The above mixed solution was treated by centrifugation (8, 000 rpm, 15 min), washing and resuspension into 460 μL of 0.1 M PBS (pH 7.4), and then the bioconjugate of aptamer-Au-Th was obtained.

### Fabrication of the antibody-aptamer sandwich assay

Before modification, the GC electrode was polished with *α*-alumina slurry (1.0, 0.3, and 0.05 μm) on a polishing cloth. Then, the electrode was sonicated in acetone and deionized water each for 10 min, and then dried under N_2_ gas. Next, 10 μL of carboxyl graphene dispersion (2 mg mL^−1^) was dropped onto the GC electrode surface. After drying by infrared lamp and washing with distilled water, the electrode was treated by activation with EDC/NHS (30 mM/2 mM) for 2 h and washed with distilled water. Subsequently, the electrode was immersed into 10 μg mL^−1^ antibody dissolved in 0.1 M PBS (pH 7.4) for 4 h. Followed by washing with 0.1 M PBS (pH 7.4), the above electrode was blocked with 10 μL of 1% BSA solution to eliminate the non-specific binding effects. After washing with PBS, the antibody-modified sensor was fabricated.

The detection of A*β* oligomers based on the antibody-aptamer sandwich strategy were performed by incubation of the prepared assay with a 10 μL of A*β* oligomers aqueous solutions with different concentrations at 37 °C. After washing with 0.1 M PBS (pH 7.4) for three times, the resultant sensor was then incubated with 10 μL of the aptamer-Au-Th bioconjugate at 37 °C. Followed by rinsing throughly with 0.1 M PBS (pH 7.4) to remove the unbound conjugate, the voltammetric responses of the sandwich assay were recorded by DPV from 0 to −0.5 V with a pulse amplitude of 0.005 V and a pulse width of 0.1 s for the detection of A*β* oligomers. The buffer solutions were purged with high purity N_2_ gas for at least 15 min prior to each electrochemical measurement.

## Additional Information

**How to cite this article**: Zhou, Y. *et al*. Fabrication of an antibody-aptamer sandwich assay for electrochemical evaluation of levels of *β*-amyloid oligomers. *Sci. Rep.* **6**, 35186; doi: 10.1038/srep35186 (2016).

## Supplementary Material

Supplementary Information

## Figures and Tables

**Table 1:** Results of the detection of A*β* oligomers in artificial CSF using the antibody-aptamer sandwich assay.

Sample no.	Sample	Added (nM)	Found (nM)	Recovery(%)
1	A*β* oligomer	1.0	0.97	97.0
2	A*β* oligomer	7.0	7.4	105.7
3	A*β* oligomer	12.0	12.3	102.5
4	A*β* oligomer	18.0	17.6	97.8
5	A*β* oligomer	23.0	23.7	103.0
