# Supplementary material for: Fabrication of an antibody-aptamer sandwich assay for electrochemical evaluation of levels of β-amyloid oligomers
Source: Sci Rep. 2016 Oct 11;6:35186. doi: 10.1038/srep35186 (PMC5057102; doi:10.1038/srep35186)
Supplement: Supplementary Information [file srep35186-s1.pdf]

Supporting Information

Fabrication of an antibody-aptamer sandwich assay for electrochemical evaluation of levels of *β*-amyloid oligomers

Yanli Zhou[1,2], Huanqing Zhang[2], Lantao Liu[1], Congming Li[2], Zhu Chang[1], Xu Zhu[1], Baoxian Ye[2] & Maotian Xu[1,2]

[1]Henan Key Laboratory of Biomolecular Recognition and Sensing, College of Chemistry and Chemical Engineering, Shangqiu Normal University, Shangqiu 476000 (P. R. China).

[2]College of Chemistry and Molecular Engineering, Zhengzhou University, Zhengzhou 450001 (P. R. China).

Correspondence and requests for materials should be addressed to L.L. (email: liult05@iccas.ac.cn) or M.X. (email: xumaotian@sqnc.edu.cn)

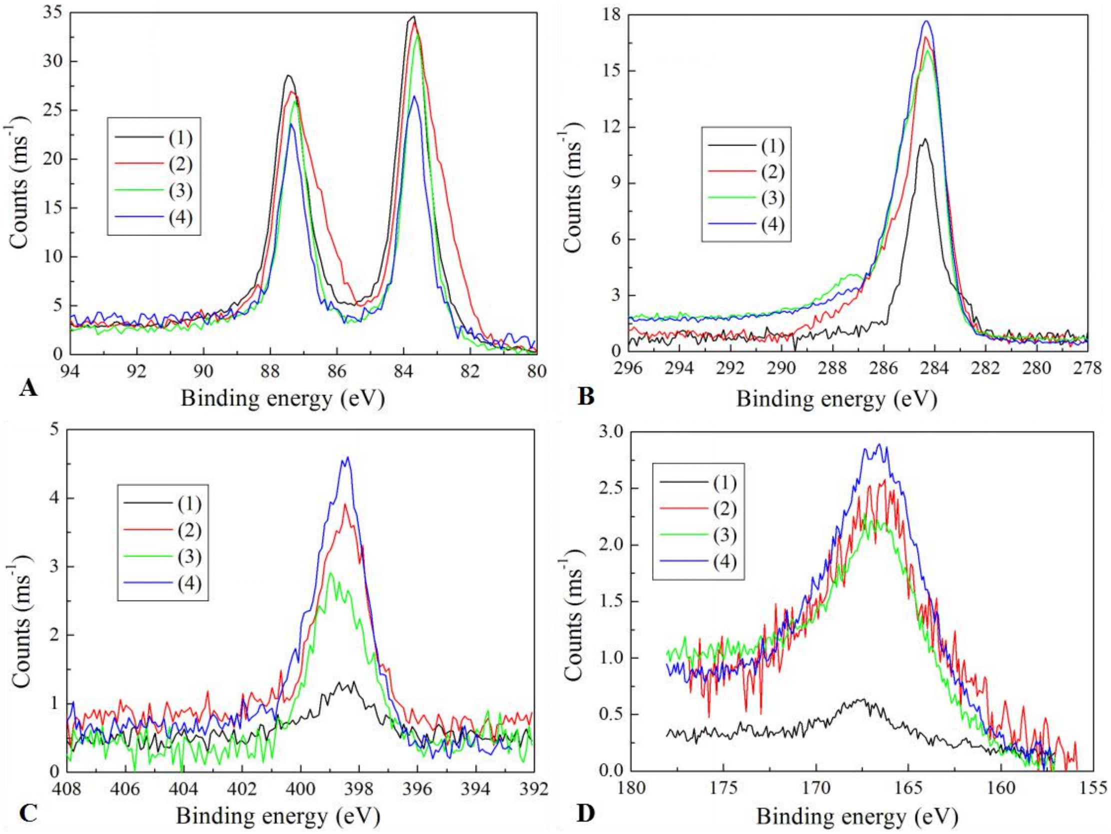

Figure S1. Au 4f (A), C 1s (B), N 1s (C), and S 2p (D) XPS spectra of AuNPs before (1) and after modification of Th (2), aptamer (3), and Th and aptamer (4).

**Method**	**Principle**	**Detection limit**	**Advantage**	**Limitation**	**Refs.**
Electrochemical biosensor	peptide recognition	0.048 nM	high sensitivity	low specificity	[7]
Electrochemical biosensor	protein recognition	0.5 pM	high sensitivity	low specificity	[8]
Electrochemical biosensor	antibody recognition	not detected	simplicity	nonspecific adsorption	[9]
Localized SPR sensor	antibody recognition	100 fM	high sensitivity and simplicity	nonspecific adsorption	[10]
SPR biosensor	antibody recognition	not detected	simplicity	nonspecific adsorption	[11]
Fluorescent sensor	interaction with probe	not detected	simplicity	low specificity	[12-16]
Fluorescent sensor	peptide recognition	0.2 nM	high sensitivity and simplicity	low specificity	[17]
Fluorescent sensor	interaction with probe	3.57 nM	high sensitivity and simplicity	low specificity	[18]
Fluorescent sensor	antibody recognition	3 pg mL^{-1}	high sensitivity and simplicity	nonspecific adsorption	[19]
Fluorescent sensor	peptide recognition	1 nM	high sensitivity and simplicity	low specificity	[20]
SERS	nanofluidic biosensor	not detected	high sensitivity and selectivity	expensive instrument, complex operation	[21]
MS	laser-induced liquid bead ion desorption	not detected	high sensitivity and selectivity	expensive instrument, complex operation	[23]
ELISA	magnetic bead immunoassay	10.7 pg mL^{-1}	high sensitivity and specificity	time-consuming operation	[29]
Electrochemical biosensor	antibody recognition	100 pM	high sensitivity and selectivity	nonspecific adsorption	This work

Table S1. Comparison of analytical characteristics for detection of Aβ oligomers using various methods.
